# Immunologic Biomarkers in Peripheral Blood of Persons With Tuberculosis and Advanced HIV

**DOI:** 10.3389/fimmu.2022.890003

**Published:** 2022-06-10

**Authors:** Artur T. L. Queiroz, Mariana Araújo-Pereira, Beatriz Barreto-Duarte, Adriano Gomes-Silva, Allyson G. Costa, Alice M. S. Andrade, João Pedro Miguez-Pinto, Renata Spener-Gomes, Alexandra B. Souza, Aline Benjamin, Flavia Sant’Anna, Marina C. Figueiredo, Vidya Mave, Padmini Salgame, Jerrold J. Ellner, Timothy R. Sterling, Marcelo Cordeiro-dos-Santos, Bruno B. Andrade, Valeria C. Rolla

**Affiliations:** ^1^ Instituto Gonçalo Moniz, Fundação Oswaldo Cruz, Salvador, Brazil; ^2^ Multinational Organization Network Sponsoring Translational and Epidemiological Research Initiative, Salvador, Brazil; ^3^ Curso de Medicina, Universidade Faculdade de Tecnologia e Ciências (UNIFTC), Salvador, Brazil; ^4^ Faculdade de Medicina, Universidade Federal da Bahia, Salvador, Brazil; ^5^ Curso de Medicina, Universidade Salvador (UNIFACS), Salvador, Brazil; ^6^ Programa de Pós-Graduação em Clínica Médica, Universidade Federal do Rio de Janeiro, Rio de Janeiro, Brazil; ^7^ Instituto Nacional de Infectologia Evandro Chagas, Fundação Oswaldo Cruz, Rio de Janeiro, Brazil; ^8^ Laboratório Interdisciplinar de Pesquisas Médicas, Fundação Oswaldo Cruz, Rio de Janeiro, Brazil; ^9^ Instituto de Pesquisa Clínica Carlos Borborema, Fundação de Medicina Tropical Doutor Heitor Vieira Dourado, Manaus, Brazil; ^10^ Division of Infectious Diseases, Department of Medicine, Vanderbilt University School of Medicine, Nashville, TN, United States; ^11^ Byramjee-Jeejeebhoy Government Medical College-Johns Hopkins University Clinical Research Site (BJGMC-JHU CRS), Pune, India; ^12^ School of Medicine, Johns Hopkins University, Baltimore, MD, United States; ^13^ Rutgers- New Jersey Medical School, Center for Emerging Pathogens, Newark, NJ, United States; ^14^ Curso de Medicina, Escola Bahiana de Medicina e Saúde Pública (EBMSP), Salvador, Brazil

**Keywords:** biomarkers, diagnosis, tuberculosis, advanced HIV, IL-15, IL-10, cytokine

## Abstract

**Introduction:**

Tuberculosis (TB) is a common opportunistic infection among people living with HIV. Diagnostic tests such as culture, Xpert-MTB-RIF, and ULTRA have low sensitivity in paucibacillary TB disease; a blood biomarker could improve TB diagnostic capabilities. We assessed soluble factors to identify biomarkers associated with TB among persons with advanced HIV.

**Methods:**

A case-control (1:1) study was conducted, with participants from Rio de Janeiro and Manaus, Brazil. People living with HIV presenting with CD4 count ≤100 cells/mm3 were eligible to participate. Cases had culture-confirmed TB (N=15) (positive for *Mycobacterium tuberculosis* [Mtb]); controls had HIV-infection only (N=15). Study visits included baseline, month 2 and end of TB therapy, during which samples of peripheral blood were obtained. A panel containing 29 biomarkers including cytokines, chemokines and growth factors was utilized to assess candidate biomarkers using Luminex technology in cryopreserved EDTA plasma samples. We used neural network analysis, based on machine learning, to identify biomarkers (single or in combination) that best distinguished cases from controls. Additional multi-dimensional analyses provided detailed profiling of the systemic inflammatory environment in cases and controls.

**Results:**

Median CD4 count and HIV-1 RNA load values were similar between groups at all timepoints. Persons with TB had lower body mass index (BMI) (median=19.6, Interquartile Range [IQR]=18.6-22.3) than controls (23.7; IQR: 21.8 = 25.5, p=0.004). TB coinfection was also associated with increased frequency of other comorbidities. The overall profile of plasma cytokines, chemokines and growth factors were distinct between the study groups at all timepoints. Plasma concentrations of IL-15 and IL-10 were on average lower in TB cases than in controls. When used in combination, such markers were able to discriminate between TB cases and controls with the highest degree of accuracy at each study timepoint.

**Conclusion:**

Among persons with advanced HIV, plasma concentrations of IL-15 and IL-10 can be used in combination to identify TB disease regardless of time on anti-TB treatment.

## Introduction

By the end of 2019, it was estimated that there were 38 million people living with HIV (PLWH) in the world, In 2020, there were still 690,000 HIV-related deaths and 1.7 million new infections globally (1). Without treatment, PLWH typically progress through several stages. When CD4 cell counts are <200 cells/mm^3^ or patients are classified as WHO stage 3 or 4, the disease is characterized as advanced, with a very high risk of death (1).

It is common for people with advanced HIV (PLWH) to experience opportunistic infections. One of the most common co-infections is tuberculosis (TB). Approximately 10 million people have had TB in 2019 globally, of whom 8% were co-infected with HIV (2). Co-infection with HIV and TB accelerates the progression of both diseases and leads to substantial deterioration of effective immune responses, significantly impacting morbidity and mortality (3–6). TB-HIV coinfection was responsible for approximately 214,000 deaths in 2020 (2).

TB diagnosis has improved recently due to the availability of rapid molecular assays such as Xpert MTB/RIF (76.5% sensitivity) and Xpert MTB/RIF ULTRA (up to 88.2% sensitivity). However, the accuracy of such diagnostic tests is still limited, particularly in PLWH, who often have paucibacillary disease and therefore negative screening for acid-fast bacilli (AFB) in sputum smears (7). In such patients, the sensitivity of ULTRA is reduced to 64.7%, and the sensitivity of Xpert MTB/RIF is 41.2% (8). On the other hand, HIV increases by at least 20-fold the risk of progression from latent *M. tuberculosis* (Mtb) infection to active TB (9). As a result, PLWH with advanced disease exhibit a high risk of incident active TB (10). Understanding how the immune response modulates Mtb infection and progression to TB disease is crucial to develop immunologic tools to identify TB in PLWH (10). Given that many immunologic proteins are regulated during Mtb infection, in the present study we tested whether quantification of such plasma molecules could serve as informative TB biomarkers in PLWH.

## Methods

### Ethical Statement

This study was approved by the Institutional Review Boards from each clinical site (CAAE: 85790218.4.1001). All participants signed an informed consent form before enrollment. All clinical and laboratory data were de-identified to protect participants’ confidentiality. All clinical investigations were conducted according to the principles of the Declaration of Helsinki.

### Study Design and Eligibility Criteria

This was a case-control study (1:1) to evaluate the performance of blood soluble molecule signatures associated with TB among PLWH with advanced disease. Cases and controls (15 participants in each group) were recruited from February 2018 to December 2019, based on study eligibility criteria. Participants were recruited if they agreed to participate, provided informed consent, were ≥18 years old, HIV-positive, and had CD4 count ≤100 cells/mm^3^. TB had to be confirmed by mycobacteriologic culture for the TB-HIV group. In the controls with HIV only, participants had no signs or symptoms of TB and were screened negative with smear and culture examination. Participants were excluded if they were pregnant or had any condition that, in the judgment of the investigator, precluded participation because it could have affected the subject’s safety. To be eligible for the TB-HIV group, TB treatment could have been initiated no more than 7 days before enrolment. Study participants were recruited from one site in Rio de Janeiro (INI- Fiocruz) and one site in Manaus (FMT), both in Brazil. These sites have active TB cohorts and affiliated HIV treatment centers.

Clinical and sociodemographic data and plasma samples were obtained at baseline, month 2 and end of therapy or equivalent timepoint in the control group (END visit). CD4 count and HIV-1 RNA (viral load; VL) were performed at all study visits (baseline, Month 2 and END of therapy). All participants (n=15, 100%) in the HIV group and 6 (40%) in the TB-HIV group were receiving antiretroviral therapy (ART) at enrollment; 9 (60%) TB-HIV patients initiated ART during TB therapy. TB-HIV patients were classified as having either pulmonary (PTB) or pulmonary + extrapulmonary (PTB+EPTB). None of the participants had only EPTB.

### Measurements of Plasma Biomarkers

All plasma samples, stored at -80°C, were quantified for 29 biomarkers (cytokines, chemokines and growth factors) using a commercially available Luminex kit (Millipore): Epidermal growth factor (EGF), EOTAXIN, Granulocyte colony-stimulating factor (G-CSF), Granulocyte-macrophage colony-stimulating factor (GM-CSF), Interferon (IFN)-α2, IFN-gamma, Interleukins (IL)-10, IL-12P40, IL-12P70, IL-13, IL-15, IL-17A, IL-1RA, IL-1a, IL-1B, IL-2, IL-3, IL-4, IL-5, IL-6, IL-7, IL-8, CXCL10, Monocyte Chemoattractant Protein (MCP)-1/CCL2, Macrophage Inflammatory Protein(MIP)-1a/CCL3, MIP-1b/CCL4, Tumor Necrosis Factor (TNF)-α, TNF-β and Vascular Endothelial Growth Factor (VEGF).

### Heatmaps

A one-sided unsupervised hierarchical clustering [Ward’s method (11)] and a heatmap representation were used to display z-score normalized log_2_-transformed data and depict the overall profile of samples and biomarkers. In this analysis, the dendrograms represent the Euclidean distance (inferring degree of similarity). This analysis was performed using the *ComplexHeatmap* R package (12). Moreover, the log_2_ fold change was measured, and the significance was calculated with the *t.test* (13) and corrected with the False Discovery Ratio (FDR) (14).

### Machine Learning Modeling

After the data preparation, we applied an averaged Neural Network (ANN) model (15), with repeated 20-fold cross-validation to classify the outcomes TB-HIV and HIV with the *caret* R package (16). The best variables were retrieved and used on a linear model for evaluation. The most informative variables were retrieved from the dataset and the classification was assessed by receiver operating characteristic (ROC) curves and the area under the curve (AUC) value ((17)).

### Network Analysis

Correlation analyses were performed using the Spearman’s rank test (18), using the rcorr function from the *Hmisc* R package (available at: https://rdrr.io/cran/Hmisc/). Only significant correlations (p<0.05) were depicted in the network. The correlation networks were constructed using the *igraph* R package (19). All analyses were performed in R (version 4.0.2). To evaluate the network reliability, we also applied bootstraps 20 with 100 replicates (20). We retrieved each network density and the degrees of each edge. The differences between the groups were tested with a t-test.

## Results

There were 15 TB-HIV cases and 15 controls with HIV. All 15 TB cases had pulmonary disease; 8 also had extrapulmonary disease: lymph node (n=1)intestinal (n=2), splenic (n=1), laryngeal (n=1), cutaneous (n=1), renal (n=1), and genitourinary (n=1). No cases of immune reconstitution inflammatory syndrome (IRIS) were diagnosed during the study.

**Figure 1:**
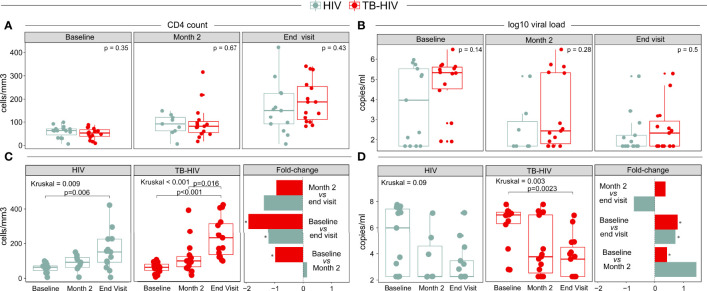
Differences in **(A, C)** CD4^+^count (cells/mm^3^) and **(B, D)** HIV viral load (HIV RNA copies/mL) between the TB-HIV and HIV groups at different timepoints. *Represents p-values < 0.001.

Cases and controls had similar clinical and demographic characteristics, though there were some differences (**Table 1**). Body mass index (BMI) was lower in TB-HIV cases (median=19.6, Interquartile Range [IQR]=18.6-22.3) than HIV controls (23.7; IQR: 21.8 = 25.5, p=0.004). However, CD4^+^ count and HIV viral load (VL) were similar between the groups at baseline and during follow-up **(** **Figures 1A, B** **)**.

**Table 1:** Characteristics of people living with advanced HIV by TB status.

Characteristics	TB-HIV (n=15)	HIV (n=15)	p-value
**Age – median (IQR)**	37.0 (30.5-38.5)	33.7 (30.2-43.2)	0.884
**Male sex – no. (%)**	10 (66.7)	12 (80.0)	0.682
**Race/Ethnicity – no. (%)**			0.337
White	2 (13.3)	5 (33.3)	
Black	3 (20.0)	2 (13.3)	
Pardo	8 (53.3)	8 (53.3)	
Indigenous	2 (13.3)	0 (0.0)	
**BMI – median (IQR)**	19.6 (18.6-22.3)	23.7 (21.8-25.5)	**0.004**
**CD4 count – median (IQR)**	53.0 (38.5-67.0)	63.0 (44.5-71.0)	0.290
**Log10 HIV Viral Load – median (IQR)**	5.33 (4.53-5.61)	5.23 (3.98-5.73)	0.113
**ART – no. (%)**	6 (40.0)	15 (100)	**0.001**
**Smoking – no. (%)**	6 (40.0)	3 (20.0)	0.427
**Passive smoking – no. (%)**	4 (26.7)	0 (0)	0.100
**Alcohol consumption – no. (%)**	13 (86.7)	12 (80.0)	1.000
**Illicit drug use – no. (%)**	4 (26.7)	2 (13.3)	0.651
**Prior TB episode– no. (%)**	3 (20.0)	0 (0.0)	0.224
**Cancer– no. (%)**	0 (0.0)	0 (0.0)	NA
**Diabetes– no. (%)**	3 (20.0)	0 (0.0)	0.224
**Renal disease– no. (%)**	0 (0.0)	0 (0.0)	NA
**Hypertension– no. (%)**	0 (0.0)	0 (0.0)	NA
**Any comorbidity– no. (%)**	15 (100)	8 (53.3)	**0.006**

Data represent no. (%), except for age and BMI, which is presented as median and interquartile range (IQR). Continuous variables were compared using the Mann-Whitney U test and categorical variables were compared using Fisher’s exact test (2x2) or Pearson’s chi-square test. Statistically significant differences (p-value < 0.05) are highlighted using bold-type font.

Definition of alcohol consumption: Past or current any consumption of alcohol. Definition of passive smoking: Living with someone who smokes. Definition of illicit drug use: Past or current illicit drug use (marijuana, cocaine, heroin or crack). Definition of Pardo ethnicity: mixture of European, black and Amerindian.

TB, tuberculosis; BMI, Body Mass Index; NA, Not applicable; ART, antiretroviral therapy.

Any comorbidity: mycosis, syphilis, retinitis, allergies.

Of note, CD4 counts increased over time in both groups (HIV: p=0.009; TB-HIV: p<0.001) (**Figure 1C**). However, the fold change in CD4^+^ count within each study group over time was different. The CD4^+^ count increase was statistically significant between baseline and month 2 in the TB-HIV group (p<0.001); and baseline *vs* END visit (p<0.001) in both groups (**Figure 1C**). The decrease in VL over time was statistically significant only in the TB-HIV group (p=0.003). The fold-change was significant when comparing baseline *vs* month 2 (p<0.001) in the TB-HIV group; and baseline *vs* END visit (p<0.001) in both groups (**Figure 1D**). Taken together, the results suggest that in PLWH, those with TB exhibited a laboratory profile indicative of more advanced disease than PLWH without TB. However, as treatment progressed, this profile became similar to that observed in the HIV control group.

**Figure 2:**
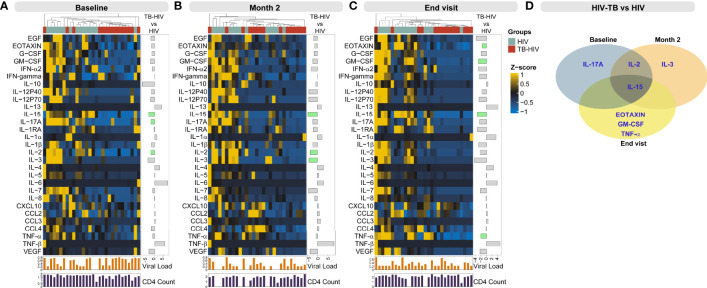
Profiling systemic inflammation to identify markers differentially expressed in persons with TB-HIV coinfection. Concentrations of the indicated biomarkers were quantified in plasma samples using Luminex technology in study participants at baseline (prior to anti-TB treatment) **(A)**, at month 2 **(B)** and at END visit **(C)** of anti-TB treatment. Data were log2-transformed, and z-score normalized to build the heatmaps at each study timepoint. A hierarchical cluster analysis (Ward’s method, with dendrograms implying the Euclidean distance) was employed to test whether combined quantification of plasma concentrations of the biomarkers could be used to segregate the TB-HIV from the HIV group. The upper colored line corresponds to the groups: in light blue are the HIV samples and in red are the TB-HIV samples. The below bar plots correspond to log10 Viral load and CD4^+^ count. The side bar plot shows the fold change values from the comparison TB-HIV vs HIV and green bars are the differences with significant false discovery rate (5%). **(D)** Venn diagram with intersection with markers that showed statistical significance when comparing TB-HIV with HIV. All significant comparisons showed a negative fold-change.

**Figure 3:**
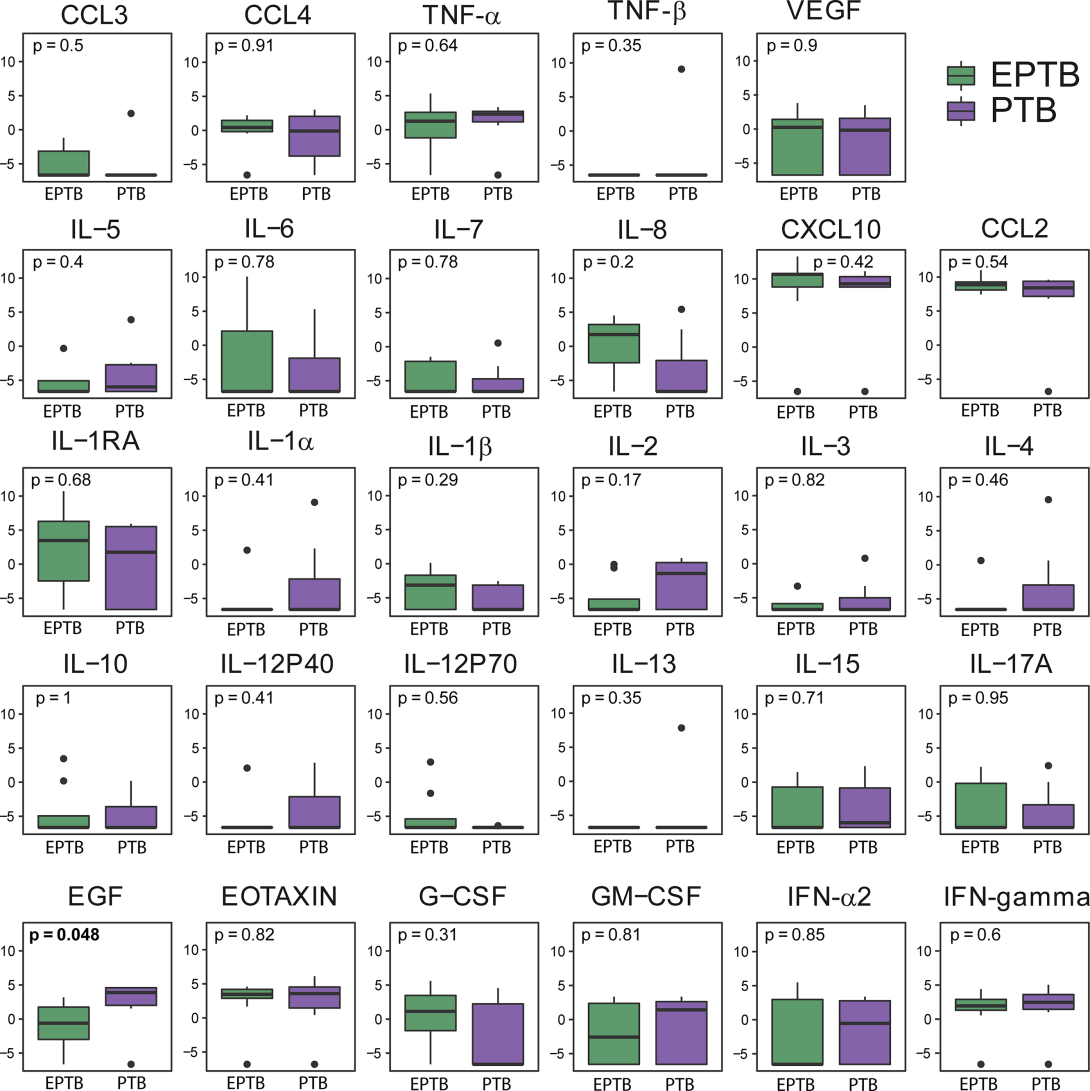
Soluble inflammatory markers do not differ according to the location of TB in TB-HIV patients. Boxplots show the levels of each marker analyzed at baseline, comparing pulmonary TB (PTB, n = 7) with PTB+extrapulmonary TB (EPTB, n = 8) in the TB-HIV population (n = 15). Only EGF showed a statistically significant difference (p<0.05) in comparisons.

**Figure 4:**
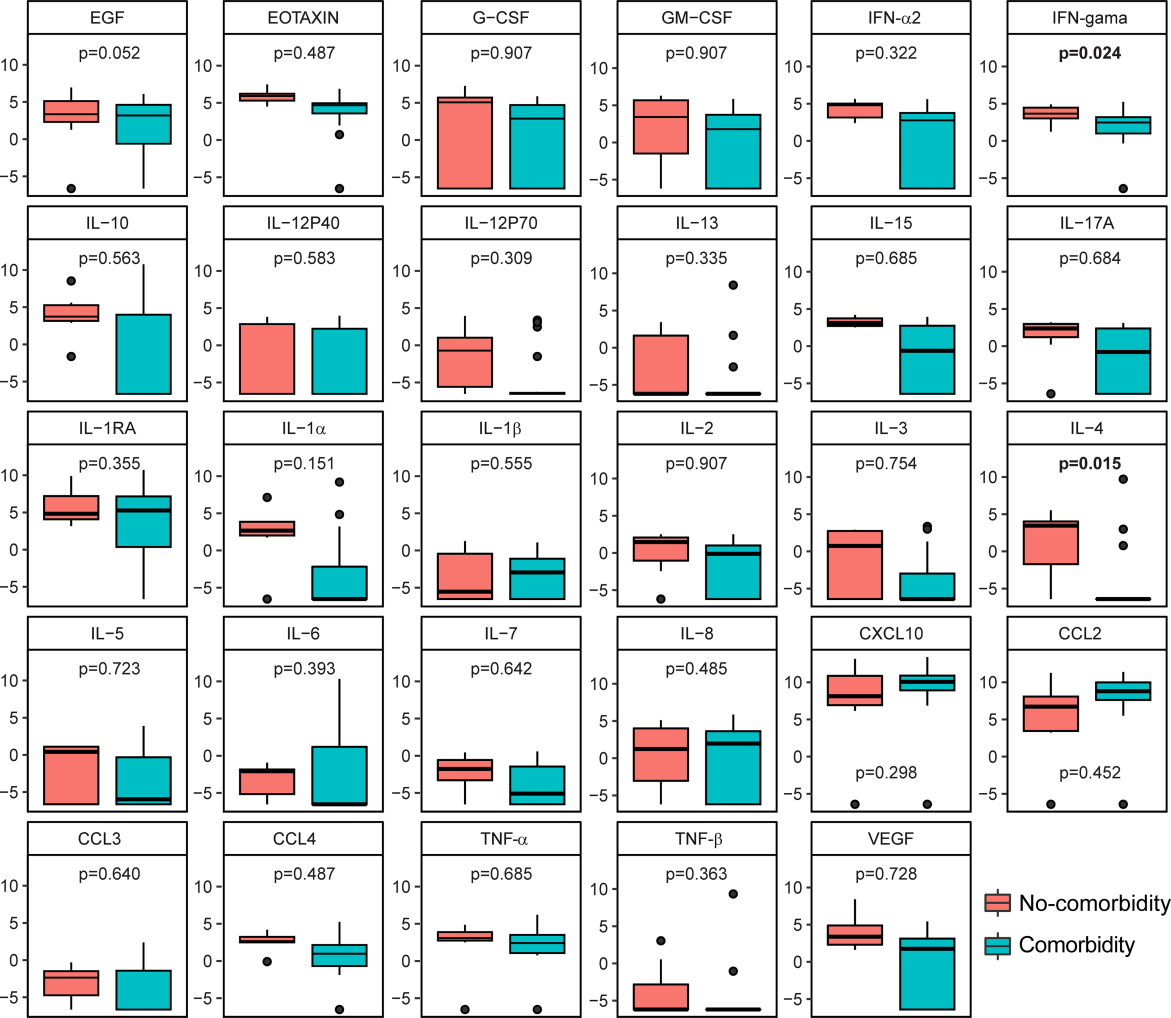
Soluble inflammatory markers differ according to comorbidity occurrence in HIV patients. Boxplots show the levels of each marker analyzed at baseline, comparing comorbidity occurrence (n = 8) with those without comorbidity (n = 7) in the HIV population (n = 15). Only IFN-gamma and IL-4 showed a statistically significant difference (p < 0.05) in comparisons.

Next, we performed multi-dimensional analyses to assess the systemic inflammatory profile of each group. First, we used an unsupervised hierarchical cluster analysis to identify inflammation profiles of distinct groups at each timepoint. It was possible to identify two main clusters at all timepoints; in general, TB-HIV patients had a distinct profile at all timepoints, with lower levels of biomarkers than HIV controls **(** **Figure 2** **).** The levels of all biomarkers measured are summarized in **Supplementary Table 1**. At baseline, this difference was significant for IL-15 (Fold Change [FC] TB-HIV vs. HIV: -3.592, p<0.001), IL-17A (FC: -2.19, p=0.044) and IL-2 (FC: -2.20, p=0.023) (**Figure 2A**). At month 2 of anti-TB therapy, this difference was significant in IL-15 (FC: -4.51, p=0.004), IL-2 (FC: -3.44, p=0.048) and IL-3 (FC: -3.98, p=0.032) (**Figure 2B**). Furthermore, at END visit, TB-HIV participants exhibited on average diminished levels of Eotaxin (FC: -1.55, p=0.0038), GM-CSF (FC: -2.89, p=0.048), IL-15 (FC: -3.45, p<0.001) and TNF-α (FC: -2.14, p<0.007) **(** **Figure 2C** **)**. We also showed the fold-change values for baseline, month 2 and END visit timepoints in the respective **Supplementary Table 3; Tables 4, 5**. IL-2 showed a significant difference between time points: baseline and month 2 while IL-15 was the only cytokine that was significantly lower at both timepoints (baseline, month 2 and END visit) (**Figure 2**). Additionally, we performed comparisons of biomarker levels in TB-HIV patients according type of TB (PTB: n=7; PTB+EPTB: n=8) and observed that there were no differences in biomarker levels between these two groups, except for EGF, which was higher in PTB (p=0.048) than in PTB+EPTB (**Figure 3**). The values of markers in each group are described in **Supplementary Table 2**. We also compared HIV patients according to comorbidities (i.e., mycosis, syphilis, retinitis, allergies, cancer, diabetes, renal disease, hypertension), and observed that those with any comorbidity presented higher values of two cytokines in comparison with those without comorbidities: IFN-gamma (Any comorbidity: 3.43, IQR:2.80-4.27; Without Comorbidity: 2.0, IQR: -2.09-2.33; p=0.024) and IL-4 (Any comorbidity: 3.23, IQR: -1.95-43.78; Without Comorbidity: -6.64, IQR:-6.64—6.63; p=0.015) (**Figure 4**).

**Figure 5:**
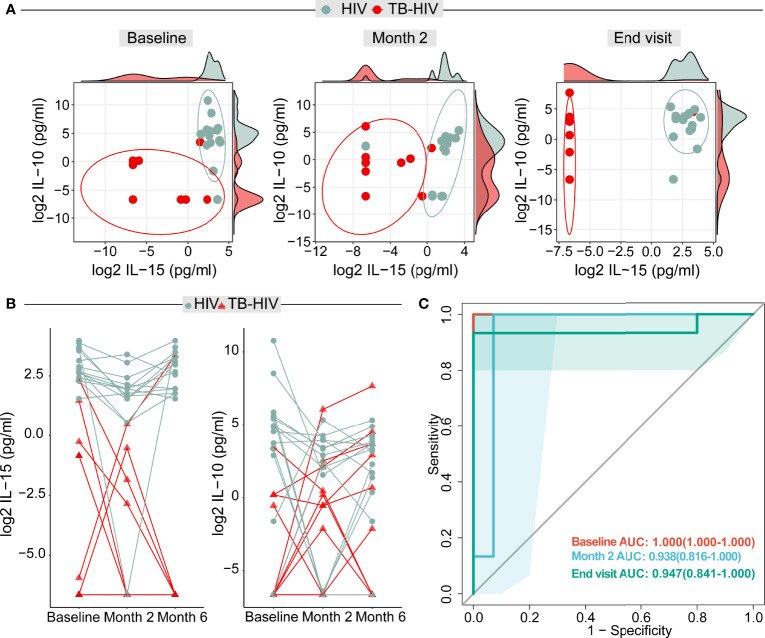
Artificial neural network analysis identified IL-15 and IL-10 as potential markers for distinguishing TB cases among persons with advanced HIV. **(A)** Dot plot from the biomarker values in both TB-HIV and HIV groups in the baseline, month 2 and END visit. The ellipses were calculated with the “distance t” from each group. The axis parallels graphs are each IL-15 and IL-10 log2 values displayed in density distribution (histograms). **(B)** Distribution of biomarkers in plasma from TB-HIV and HIV participants: Dots represent individuals with HIV-infection, whereas the triangles indicate TB-HIV participants. Values from a given study participant at the indicated study timepoints are connected through colored lines. The y axis shows the log2 IL-15 and IL-10 values. **(C)** Receiver Operator Characteristics (ROC) analysis using the plasma concentrations of both IL-15 and IL-10 at each study timepoint was performed to test the accuracy of the combined markers in distinguishing TB-HIV from HIV cases. All the areas under the curves (AUC) exhibited p-value of <0.0001.

Next, we used neural network analysis, an approach based on machine learning (16), to identify the marker or combination of markers that could be more informative to distinguish cases from controls. Using this approach, we identified IL-15 and IL-10 as the best classifiers to distinguish groups at all the study timepoints (**Figure 5**). The thresholds were 5.13 pg/ml for IL-15 and 1.14 pg/ml for IL-10. Indeed, the distribution of these biomarkers could fairly classify the samples from each group at baseline, month 2 and END visits (**Figure 5A**). We also verified the individual variation of IL-15 and IL-10 at the different timepoints and found that although the concentrations of both biomarkers were substantially diverse in the study population, TB-HIV participants in general exhibited lower concentrations throughout the entire study period (**Figure 5B**). The best classifiers identified in the neural network analysis were retrieved and we tested their classification performance at the different timepoints using ROC curves. Using this approach, we identified that the cytokines showed better performance at baseline (area under the curve [AUC]: 1.0, p<0.0001), followed by a slight decrease in performance at month 2 (AUC: 0.964, p<0.0001) and a further decrease at END visit (AUC: 0.950, p<0.0001) (**Figure 5C**).

**Figure 6:**
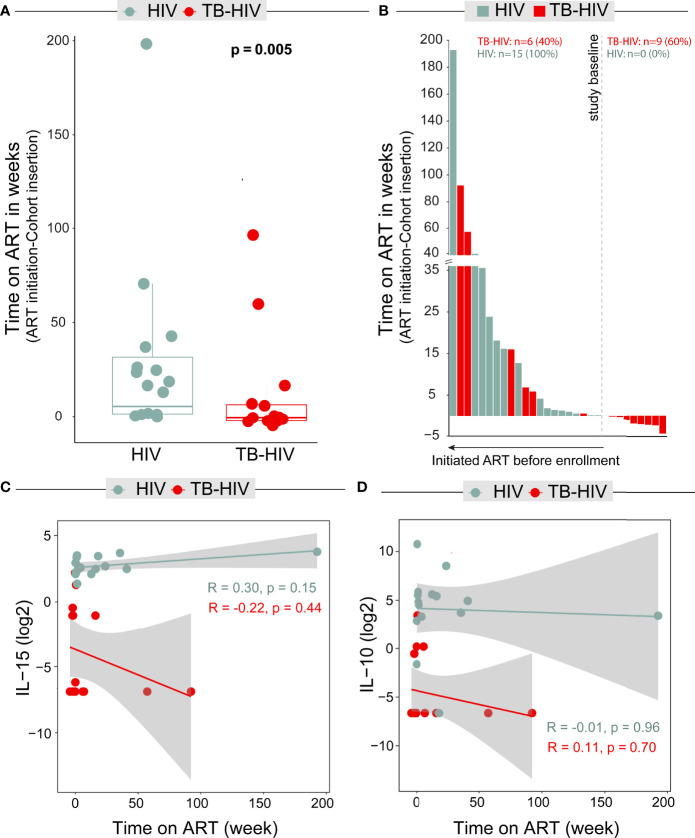
Timing of antiretroviral therapy (ART) does not impact IL-15 and IL-10 levels. **(A)** Boxplot shows that those with TB-HIV had a shorter time between initiation of ARV and enrollment in the study in relation to HIV patients (Mann-Whitney test p = 0.005). **(B)** Bar graph shows that 8/15 (53.3%) TB-HIV started ARV before being enrolled. Figures **(C, D)** show linear regressions between ART time and IL-15 and IL-10 levels, respectively. A regression was performed for each group (TB-HIV- red and HIV - blue) in relation to each of the biomarkers. No statistically significant differences were found in any of the markers or groups.

We observed that time on ART was significantly different between the groups (p=0.005), The HIV control group had a longer time on ART (4.14; IQR:1.14-21 weeks) compared to TB-HIV cases (-0.14, IQR: 1.92-6.36 weeks) (**Figure 6A**). At study initiation, the entire HIV control group was already on ART, and only 6 of the TB-HIV group were on ART. Nine of 15 patients in the TB-HIV group started ART after enrollment (**Figure 6B**). To ensure that time on ART was not influencing the levels of these biomarkers, we performed linear regressions with both measures (time on ART versus IL-15 and time on ART versus IL-10), which demonstrated that there were no significant correlations between time on ART and the levels of such cytokines in peripheral blood (**Figures 6C, D**).

**Figure 7:**
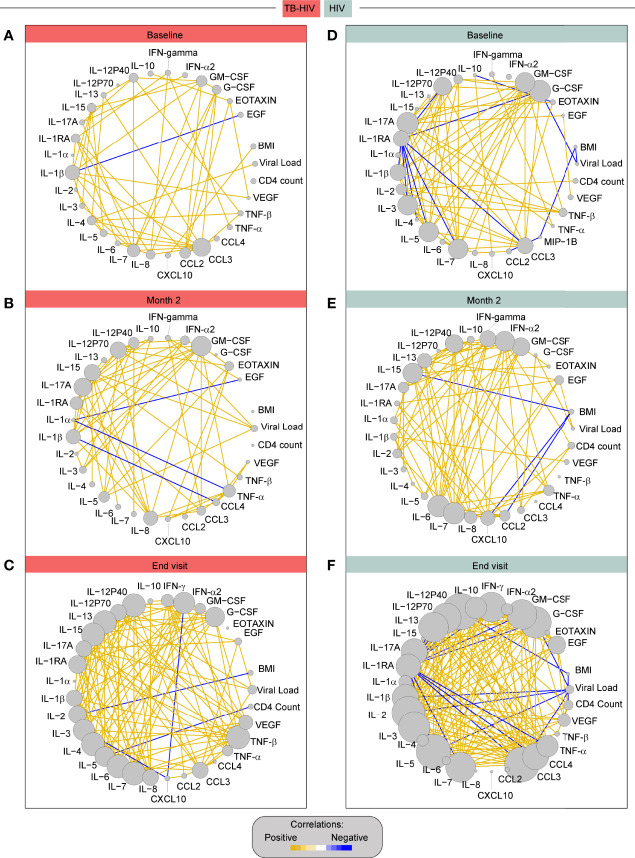
Correlation network of TB-HIV and HIV groups across the timepoints. The diameter of each circle is proportional to the number of significant correlations prospectively. The connecting lines represent statistically significant correlations (P < 0.05). Yellow connecting lines represent positive correlations while blue lines infer negative correlations. **(A)** – TB-HIV group at baseline, **(B)** – TB-HIV group at month 2, **(C)** – TB-HIV group at END, **(D)** –HIV group at baseline, **(E)** – HIV group at month 2, and **(F)** – HIV group at end visit.

To understand the coordination of immune responses in the peripheral blood (21) we used network analysis to assess correlations between the concentrations of the biomarkers, CD4 counts, VL, and BMI. This analysis revealed that the network profiles of the different clinical groups at each study timepoint were very distinct (**Figure 7**). At month 2, VL was positively correlated with IFN-gamma , IL-12p70, IL-15 and IL-5 (**Figure 7**). At the END visit, CD4 counts were negatively correlated with IL-5. VL was positively correlated with EGF, IL-12p70, IL-17a and CCL3. BMI was negatively correlated with IL−2 (**Figure 7**). In the HIV-control group, VL was negatively correlated with BMI and G-CSF count, at baseline (**Figure 7**). At month 2, VL was positively correlated with G−CSF. BMI was negatively correlated with IL−15, CXCL10 and CCL2 (**Figure 7**). At the END visit, VL was negatively correlated with IL-1B, IL-2, IL-5, CCL3, G−CSF and BMI. CD4 count was negatively correlated with BMI and positively correlated with EGF and VEGF **(** **Figure 7**).

**Figure 8:**
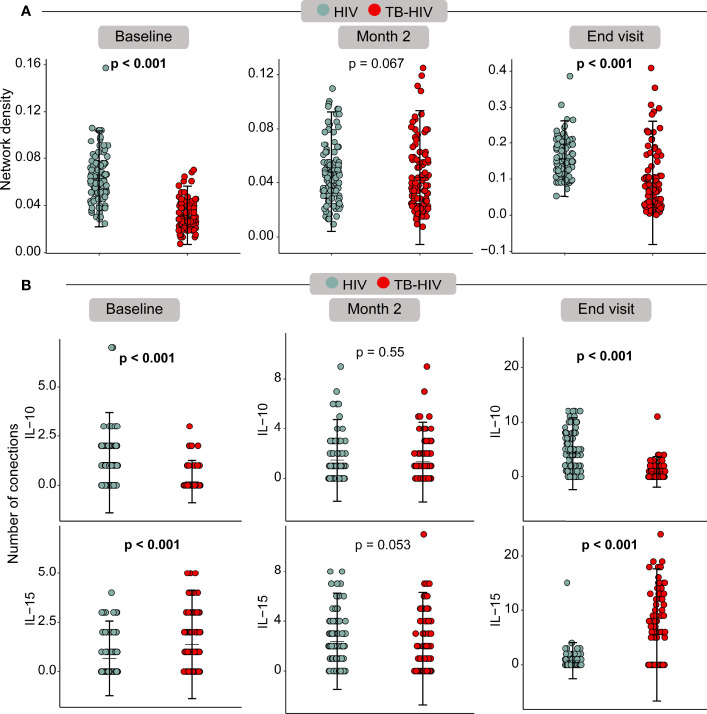
Bootstrap correlation network analysis showed differences between baseline and END visits. **(A)** – Network density across the timepoints; **(B)** – Edge degree from IL-15 and IL-10 across the baseline, month 2 and end visit.

The HIV control group had significantly higher network density than TB-HIV cases at baseline (p<0.001) and at the END visit (p<0.001) (**Figure 8A**). Moreover, the IL-10 number of connections was higher in the HIV group compared with TB-HIV at the baseline (p<0.001) and END visits (p<0.001) (**Figure 8B**). Nevertheless, the IL-15 degree was significantly increased in TB-HIV at baseline (p<0.001) and END visits (p<0.001), suggesting that this molecule played a crucial role in the shift of network density between the groups, and regulating the immune response on the TB-HIV group (**Figure 8B**).

## Discussion

This is a study conducted in PLWH and advanced immunosuppression in both TB-HIV and HIV-infected participants. TB is an opportunistic infection (OI) that contributes to HIV replication and harm, being one of the most prevalent OI in the world and responsible for most deaths in HIV-infected population (22). As it is difficult to diagnose TB in advanced HIV due to the low sensitivity of diagnostic tests and X-rays. Moreover, signs and symptoms of TB are common to other OI and delays to diagnosis and treatment of TB can lead to hospitalization, death and other OI diagnosis. In this study we wanted to identify a biomarker that could segregate advanced HIV with TB from those without TB, using baseline characteristics, VL, CD4 count, and plasma soluble factors at baseline and follow up visits.

Our study found a lower BMI in the TB-HIV group showing the impact of weight loss to cellular immune response (23) that increase the risk to develop TB (24). Other comorbidities were more frequently seen in the TB-HIV group as well (p=0.006) which could increase morbidity and mortality. During follow-up CD4 counts were similar in both groups as well as VL although the reduction of VL was evident when comparing baseline values *vs* month 2 (p<0.001) in TB-HIV group; and baseline *vs* end visit (p<0.001) in both groups. Our results suggest that in PLWH, TB diagnosis is associated with a laboratory profile indicative of more advanced disease than PLWH without TB. However, the TB-HIV group seems to gradually reach a similar profile observed in HIV-infected group.

Alternative assays for TB diagnosis should be recommended in cases of low sensitivity to detect Mtb by bacteriologic or molecular assays. In the case of PLWH approaches based on detection of blood proteins to diagnose TB have been already evaluated (10, 25). The search for an immunologic biomarker detected directly in plasma, and not after the (often overnight) stimulation assays, has the advantage to become a potential point-of-care rapid diagnostic test (26).

In the present work the two-marker host biosignature based on IL-15 and IL-10 plasma levels were able to define at baseline TB among PLWH with CD4 ≤100 cells/mm^3^. Another work by Verma and colleagues (2018) done in Uganda founded plasma IFN-gamma as a good TB classifier (AUC 0.98) among PLWH (CD4 ≤100 cells/mm^3^) (10). It was interesting to note that IFN-gamma was not among plasma cytokines profile able to classify TB in the present Brazilian PLWH groups. The genetic background of different populations possibly directly interferes with the ability to produce cytokines and reinforces the requirement for validation of potential biomarkers in different ethnicities. Another seven-marker (ApoA-1, CFH, CRP, IFN- gamma, CXCL10, SAA, and transthyretin) serum protein biosignature for the diagnosis of TB, irrespective of HIV-infection status or ethnicity in Africa, were identified as a promising biomarker for field friendly point-of-care screening test for pulmonary TB (27). However, tests based on large biosignatures are expensive and complex to be manufactured, making it difficult to use them as a point of care in the future (28). In a single protein approach, Yoon and colleagues (2017) have proposed for PLWH with CD4 <340 cells/mm^3^ the quantification of C-reactive protein (CRP) by finger-prick blood sample, presenting 94% of sensitivity and 72% of specificity (25). Also, Lesosky and colleagues (2019) discussed the possibility to use plasma biomarkers based on immune response (IL-2 and IFN-gamma) as a predictive tool for TB progression among PLWH (29).

Although the present signature presents 100% of sensitivity and 100% of specificity it is not known whether non Mtb infection or other OI in association with HIV-infection could stimulate a similar cytokine profile and interfere with the specificity of this biomarker based on immunologic molecules. Also, this ability to classify TB into PLWH was maintained over time of TB treatment (month 2 and END visit) and is necessary to assess how long this signature will be maintained post treatment, since a false positive result could configure a limitation of this biomarker to identify active TB in PLWH with a previous history of TB treatment. As described before, diagnosis of TB patients with a previous history of TB are considered a challenge when using biosignatures based on the dosage of soluble blood factors (28).

The immune system coordination accessed by a network analysis of spearman correlations is an interesting approach to help understand biomarkers (30) that could be useful to classify TB among PLWH with advanced immunosuppression. At baseline it was clearly observed the lower network density by TB-HIV group in comparison to the HIV group. Coinfection with TB in PLWH probably imprints on the immune system a dysfunction of innate and adaptive immune cells that result in a significant dysregulation in cytokine production. At month 2 after TB treatment these differences between cases and controls disappear, which could be attributed by the Mtb control and the beginning recovery of the immune system homeostasis. At the END visit, the density network returned to be statistically higher in the HIV group in comparison to TB-HIV group, but specially for IL-15, the higher number of correlation interactions in TB-HIV group is suggestive that this cytokine has an important role in restoring the balance of the immune system after Mtb control.

Our study has limitations. This was an exploratory study with a small sample size with patients with only CD4 ≤100 cells/mm^3^. Thus, additional comparisons exploring the potential interference of OIs in the performance of the discrimination model presented here were hampered due to underpowered statistical analysis. Thus, additional studies are warranted to define the relationships between OI and diagnosis performance of the proposed biosignature. Regardless, this study presents an pioneering approach that targets clinical and plasma immunological biomarkers for PLWH with advanced disease, and its results bring new insights that could be validated in other populations especially in other regions with distinct genetic and/or epidemiologic settings.

In conclusion we found in the present work that the combination of IL-15 and IL-10 plasma levels was able to define at baseline a biomarker signature associated with TB in PLWH and advanced immunosuppression. Although this test presents 100% of sensitivity and 100% of specificity this is an exploratory study with preliminary finding. Our results should be validated in bigger cohorts and other races.

## Data Availability Statement

The raw data supporting the conclusions of this article will be made available by the authors, without undue reservation.

## Ethics Statement

The studies involving human participants were reviewed and approved by Institutional Review Board of the Instituto Nacional de Infectologia, Fundação Oswaldo Cruz (CAAE: 85790218.4.1001). The patients/participants provided their written informed consent to participate in this study.

## Author Contributions

AQ performed the analyses and wrote the first draft; MA-P Performed the analyses with AQ, integrated clinical and immunologic data and wrote the first draft; BB-D and AA performed the luminex experiment, curated the luminex output data, helped in data analysis and design of figures and wrote the first draft; AG-S and AC, data interpretation and wrote the manuscript discussion; RS-G and AS clinical team from Manaus; AB and FS clinical team from Rio de Janeiro; VM, PS, and JE India team; TS, MC, BA, and VR established the initial concept and wrote the manuscript. JE, PS, and VM established the initial concept and revised the manuscript. All authors contributed to the article and approved the submitted version.

## Funding

The study was funded by CRDF Global (DAA3-17-63158-1/BRAZIL). BA is supported by the Intramural Research Program of the Oswaldo Cruz Foundation, Brazil. MA-P received a fellowship from Coordenação de Aperfeiçoamento de Pessoal de Nível Superior (finance code 001). BA and VR are senior fellows from The Brazilian National Council for Scientific and Technological Development, Brazil.

## Conflict of Interest

The authors declare that the research was conducted in the absence of any commercial or financial relationships that could be construed as a potential conflict of interest.

## Publisher’s Note

All claims expressed in this article are solely those of the authors and do not necessarily represent those of their affiliated organizations, or those of the publisher, the editors and the reviewers. Any product that may be evaluated in this article, or claim that may be made by its manufacturer, is not guaranteed or endorsed by the publisher.
