# Supplementary material for: Immunologic Biomarkers in Peripheral Blood of Persons With Tuberculosis and Advanced HIV
Source: Front Immunol. 2022 Jun 10;13:890003. doi: 10.3389/fimmu.2022.890003 (PMC9226490; doi:10.3389/fimmu.2022.890003)
Supplement: Supplementary file 1 [file DataSheet_1.pdf]

Immunologic Biomarkers in Peripheral Blood of persons with Tuberculosis and advanced HIV

Artur T L Queiroz. Mariana Araújo-Pereira. Beatriz Barreto-Duarte. Adriano Gomes-Silva. Allyson G Costa. Alice M.S. Andrade. João Pedro Miguez-Pinto. Renata Spener-Gomes. Alexandra B Souza. Aline Benjamin. Flavia Sant'Anna. Marina C Figueiredo. Vidya Mave. Padmini Salgame. Jerrold J Ellner. Timothy R Sterling. Marcelo Cordeiro dos Santos. Bruno B. Andrade. and Valeria C Rolla

Supplementary Material

Content

1. Supplementary Table 1
2. Supplementary Table 2
3. Supplementary Table 3
4. Supplementary Table 4
5. Supplementary Table 5

Supplementary Tables:

Supplementary Table 1. Description of biomarker summary statistics in the groups of the study. (The table was provided in XLS separately)

Table note: Abbreviations: TB: Tuberculosis; HIV: Human Immunodeficiency Virus.

Supplementary Table 2. Description of biomarker summary statistics according to the form of tuberculosis.

Biomarker	Type	Mean	Standard Deviation
EGF	EPTB	-0.61	3.82
EGF	PTB	3.88	4.06
EOTAXIN	EPTB	3.62	3.77
EOTAXIN	PTB	3.73	4.32
G-CSF	EPTB	1.12	4.81
G-CSF	PTB	-6.64	5.37
GM-CSF	EPTB	-2.65	4.84
GM-CSF	PTB	1.34	4.90
IFN-a2	EPTB	-6.64	5.53
IFN-a2	PTB	-0.64	4.79
IFN-gamma	EPTB	1.92	3.38
IFN-gamma	PTB	2.45	3.86
IL-10	EPTB	-6.64	4.01
IL-10	PTB	-6.64	3.16
IL-12P40	EPTB	-6.64	3.08
IL-12P40	PTB	-6.64	4.52
IL-12P70	EPTB	-6.64	3.60
IL-12P70	PTB	-6.64	0.10
IL-13	EPTB	-6.64	0.00
IL-13	PTB	-6.64	5.52
IL-15	EPTB	-6.64	3.56
IL-15	PTB	-5.94	3.74
IL-17A	EPTB	-6.64	3.86
IL-17A	PTB	-6.64	3.90
IL-1RA	EPTB	3.47	6.58
IL-1RA	PTB	1.76	6.21
IL-1α	EPTB	-6.64	3.08
IL-1α	PTB	-6.64	6.33
IL-1β	EPTB	-3.06	2.72
IL-1β	PTB	-6.64	2.03
IL-2	EPTB	-6.64	2.94
IL-2	PTB	-1.35	3.62

IL-3	EPTB	-6.64	1.58
IL-3	PTB	-6.64	2.92
IL-4	EPTB	-6.64	2.54
IL-4	PTB	-6.64	6.24
IL-5	EPTB	-6.64	2.93
IL-5	PTB	-5.97	3.88
IL-6	EPTB	-6.64	6.71
IL-6	PTB	-6.64	5.37
IL-7	EPTB	-6.64	2.49
IL-7	PTB	-6.64	2.81
IL-8	EPTB	1.71	4.46
IL-8	PTB	-6.64	5.23
CXCL10	EPTB	10.55	6.23
CXCL10	PTB	9.15	6.22
CCL2	EPTB	9.02	1.13
CCL2	PTB	8.56	5.88
CCL3	EPTB	-6.64	2.40
CCL3	PTB	-6.64	3.41
CCL4	EPTB	0.33	2.75
CCL4	PTB	-0.19	4.04
TNF-α	EPTB	1.26	4.45
TNF-α	PTB	2.29	3.47
TNF-β	EPTB	-6.64	0.00
TNF-β	PTB	-6.64	5.87
VEGF	EPTB	0.36	4.38
VEGF	PTB	-0.07	4.62

Table note: Abbreviations: PTB: Pulmonary Tuberculosis; EPTB: Extrapulmonary Tuberculosis.

Supplementary Table 3. Fold-change values of the biomarkers in the baseline timepoint.

Biomarker	FC	FDR
EGF	-2.11	0.34
EOTAXIN	-1.73	0.07
G-CSF	-1.65	0.95
GM-CSF	-2.64	0.15
IFN-α2	-1.50	0.50
IFN-gamma	-0.04	1.00
IL-10	-7.44	1.00
IL-12P40	-1.98	0.78
IL-12P70	-2.42	1.00
IL-13	4.00	1.00
IL-15	-3.60	0.00
IL-17A	-2.19	0.04
IL-1RA	-0.39	1.00
IL-1α	1.38	1.00
IL-1β	-1.78	0.97
IL-2	-2.20	0.02
IL-3	-4.03	0.34
IL-4	2.97	1.00
IL-5	0.12	1.00
IL-6	7.75	1.00
IL-7	-1.15	1.00
IL-8	-0.15	1.00
CXCL10	-0.03	1.00
CCL2	0.32	1.00
CCL3	0.33	1.00
CCL4	-2.02	0.50
TNF-α	-1.12	1.00
TNF-β	5.97	1.00
VEGF	-3.40	1.00

Table note: Abbreviations: FC: Fold-change; FDR: False discovery rate.

Supplementary Table 4. Fold-change values of the biomarkers in the month 2 timepoint.

Biomarker	FC	FDR
EGF	-1.66	1.00
EOTAXIN	-1.32	1.00
G-CSF	-1.56	1.00
GM-CSF	-2.13	0.20
IFN-α2	-3.00	0.53
IFN-gamma	0.03	1.00
IL-10	-0.79	1.00
IL-12P40	-3.99	0.40
IL-12P70	-4.02	0.10
IL-13	2.02	1.00
IL-15	-4.52	0.00
IL-17A	-2.12	0.53
IL-1RA	-1.96	0.73
IL-1α	2.28	1.00
IL-1β	-2.16	0.53
IL-2	-3.44	0.05
IL-3	-3.99	0.03
IL-4	2.94	1.00
IL-5	0.54	1.00
IL-6	3.05	1.00
IL-7	-4.24	1.00
IL-8	-1.24	1.00
CXCL10	0.64	1.00
CCL2	0.99	1.00
CCL3	1.71	1.00
CCL4	0.74	1.00
TNF-α	-0.79	1.00
TNF-β	7.92	1.00
VEGF	-3.74	0.53

Table note: Abbreviations: FC: Fold-change; FDR: False discovery rate.

Supplementary Table 5. Fold-change values of the biomarkers in the end visit timepoint.

Biomarker	FC	FDR
EGF	-3.76	0.19
EOTAXIN	-1.55	0.04
G-CSF	-1.95	0.56
GM-CSF	-2.89	0.05
IFN-α2	-2.85	0.19
IFN-gamma	-2.53	0.44
IL-10	0.50	1.00
IL-12P40	-1.72	1.00
IL-12P70	-2.71	0.51
IL-13	4.19	1.00
IL-15	-3.44	0.00
IL-17A	-2.46	0.23
IL-1RA	-3.29	0.21
IL-1α	3.50	1.00
IL-1β	-2.81	0.37
IL-2	-2.32	0.33
IL-3	-4.46	0.28
IL-4	2.55	1.00
IL-5	0.09	1.00
IL-6	3.89	1.00
IL-7	-2.24	0.45
IL-8	-0.24	1.00
CXCL10	0.22	1.00
CCL2	0.37	1.00
CCL3	0.64	1.00
CCL4	-0.25	1.00
TNF-α	-2.14	0.01
TNF-β	4.95	1.00
VEGF	-2.40	0.48

Table note: Abbreviations: FC: Fold-change; FDR: False discovery rate.
